# Translating bone marrow rejuvenation from the bench to beside

**DOI:** 10.18632/aging.204142

**Published:** 2022-06-21

**Authors:** Faisal J. Alibhai, Ren-Ke Li

**Affiliations:** 1 Toronto General Hospital Research Institute, University Health Network, Toronto, Ontario, Canada; 2 Division of Cardiac Surgery, University Health Network and University of Toronto, Toronto, Ontario, Canada

**Keywords:** aging, bone marrow, rejuvenation, myocardial infarction, stem cells, inflammation

As we age the function of hematopoietic stem cells (HSCs) declines due to changes in the local stem cell niche and the accumulation of intrinsic cell damage. Impaired HSC function in-turn contributes to a decline in immune cell function which leads to the accumulation of senescent cells, chronic inflammation, and reduced responses to injury. Using pre-clinical models our group has demonstrated that transplant of young bone marrow (BM) HSCs into aged recipients repopulates the aged BM with young functional stem cells and improves tissue repair responses in the aged heart, skeletal muscle, brain, and retina [1]. However, considerably less is known about how aging impacts human BM cell responses to injury, thus our group recently sought to expand on our pre-clinical studies using a human xenograft transplant model [2].

Sternal BM was collected from middle aged (56.4±0.97yrs) and old patients (72.7±0.59yrs), after which CD34^+^ cells were isolated and transplanted into young NOD-scid-IL2rγnull (NSG) mice. Three months after BM transplant (BMT) we assessed the reconstitution potential of these cells and their ability to participate in infarct healing following myocardial infarction (MI). Human CD34^+^ cells exhibited an age-dependent decline in colony formation in-vitro and lymphoid cell production in-vivo, consistent with past studies. Following MI, animals reconstituted with CD34^+^ cells exhibited significant age-dependent differences in our functional outcome measurements. Mice reconstituted with middle aged CD34^+^ cells exhibited less LV dilation and infarct expansion as well as better preserved cardiac function compared with those reconstituted with CD34^+^ cells from aged patients. Further examination of injury responses revealed that the age of CD34^+^ cells transplanted impacted both donor (human) and host (mouse) immune cell infiltration into the heart post-MI. Animals reconstituted with middle aged CD34^+^ cells exhibited the expected temporal immune response with a greater number of CD45^+^ cells in the heart at 3 days post-MI followed by a decline by 7 days post-MI. In contrast, animals reconstituted with old CD34^+^ cells exhibit a blunted immune response, primarily driven by a lower human T-cell response as well as reduced mouse monocyte and neutrophil infiltration into the heart at 3 days post-MI. Collectively our findings indicate that BM from aged patients produce cells that are less capable of participating in cardiac repair and also adversely affect host immune cell responses post-MI [2]. Moreover, these data support the notion that BM rejuvenation may improve tissue repair in aged patients.

Replacement of dysfunctional aged BM with functional young BM cells has potential to be an effective rejuvenation approach which can restore tissue repair responses in multiple organ systems. Recent studies in mice have demonstrated that young HSCs are resistant to pro-aging signals in the aged environment [3], suggesting that transplanted young cells can maintain their function over a prolonged period when transplanted into aged individuals. There are of course many challenges that need to be overcome to successfully translate this type of therapy for clinical use. First, older patients in general have reduced tolerance to BMT and historically patient age has been a barrier in the decision to proceed with transplantation. Interestingly, registry analyses indicate that BMT for the treatment of malignant hematopoietic diseases has increased in recent years in older patients, possibly to due advancements in associated transplant screening and therapy [4,5]. However, despite increased use the procedures associated with BMT have adverse effects, especially in patients receiving allogeneic transplants [6]. The development of minimally toxic methods to transplant BM cells into aged recipients will be essential for the use of BMT as a rejuvenation therapy. One development in this area involves the use of antibodies (e.g. anti-CD47 and anti-c-Kit) to deplete the host BM cells; this method was previously shown to facilitate high level autologous and allogenic donor cell engraftment in non-immunodeficient mice [7]. Although long term toxicity has not been evaluated, development of these alternative approaches could help facilitate BMT with a lower toxicity profile. It is also possible that complete replacement may not be necessary and that a mixed chimerism of young/old cells may be sufficient to yield beneficial effects. This could allow for a lower dose of BM ablation procedures. Second, the need for donor-recipient matching imposes a supply issue on the number cells available for transplantation. This may be overcome by advances in stem cell technologies, whereby the development of universal donor lines or recipient matched lines could provide a supply of young HSCs which can be readily transplanted into aged individuals [8]. Alternatively, banking a patient’s HSCs at a younger age or ex-vivo rejuvenation of an aged patient’s HSCs using approaches such as partial reprogramming may bypass the need for donor matching, as the aged patient’s own cells could be collected and retransplanted. Lastly, there may also be a need to rejuvenate the thymus and/or secondary lymphoid structures. Given that BM cells closely interact with these tissues and that their structure/function changes with aging, BM rejuvenation may only yield partial results without targeting of these additional structures. Aside from our transplant approach, pharmacological based therapies have also been shown to rejuvenate aged animals. However, it is not clear how much of the decline in tissue repair is due to environmental changes, such as those mediated by the senescent cell secretome, vs. the accumulation of intrinsic cell defects. Combined pharmacological and cell replacement approaches may prove to be more effective than either therapy alone. In summary, pre-clinical studies have demonstrated that aging affects mouse and human BM response to injury and that BM rejuvenation using BMT can benefit multiple organ systems. Although much work is needed to translate BMT to the clinic for rejuvenation, this approach holds great potential as a rejuvenation therapy.
